# Ultrasonography in the emergency department

**DOI:** 10.1186/s13054-016-1399-x

**Published:** 2016-08-15

**Authors:** Micah R. Whitson, Paul H. Mayo

**Affiliations:** Hofstra Northwell School of Medicine, Long Island Jewish Medical Center, 270-05 76th Avenue, New Hyde Park, NY 11040 USA

**Keywords:** Ultrasound, Emergency medicine, Critical care

## Abstract

Point-of-care ultrasonography (POCUS) is a useful imaging technique for the emergency medicine (EM) physician. Because of its growing use in EM, this article will summarize the historical development, the scope of practice, and some evidence supporting the current applications of POCUS in the adult emergency department. Bedside ultrasonography in the emergency department shares clinical applications with critical care ultrasonography, including goal-directed echocardiography, echocardiography during cardiac arrest, thoracic ultrasonography, evaluation for deep vein thrombosis and pulmonary embolism, screening abdominal ultrasonography, ultrasonography in trauma, and guidance of procedures with ultrasonography. Some applications of POCUS unique to the emergency department include abdominal ultrasonography of the right upper quadrant and appendix, obstetric, testicular, soft tissue/musculoskeletal, and ocular ultrasonography. Ultrasonography has become an integral part of EM over the past two decades, and it is an important skill which positively influences patient outcomes.

## Background

This article aims to review the historical development, organizational support, scope of practice, and evidence supporting applications of ultrasonography in the emergency department. A literature search and review was performed on PubMed and via the Hoftra Northwell School of Medicine medical library.

## Main text

### Introduction

Point-of-care ultrasonography (POCUS) is a useful imaging technique for the emergency medicine (EM) physician. Comprehensive training in POCUS is presently a mandatory part of EM training in North America, and is used extensively by some EM teams in Europe. Because of its growing use in EM, this article will summarize the applications of POCUS in the emergency department. The review will be limited to the use of EM POCUS in the adult patient. Because of space constraints, the article will not provide instruction on the clinical aspects of image acquisition, image interpretation, or the cognitive base of the field; these are well presented in standard textbooks and literature on the subject. Instead, the focus will be on reviewing the scope of practice of EM POCUS.

### Consultative ultrasonography versus POCUS

Standard consultative ultrasonography requires the frontline EM physician to order the examination and to rely on the radiologist or cardiologist to perform it in a timely and clinically relevant manner. In using POCUS, the EM physician performs all image acquisition and interpretation at the point of care and uses the information immediately to address specific hypotheses and to guide ongoing therapy. This requires that the EM physician has skill at image acquisition, image interpretation, and the cognitive elements required for immediate application of the results of the examination. The frontline EM clinician has full knowledge of the case, and is able to rapidly integrate the results of the POCUS examination into the management plan, whereas the traditional consultative model involves delay in performance of the study, delay in its interpretation, and delay in transmission of the results to the clinical team. In addition, the radiology or cardiology consultant is not fully aware of the clinical facts of the case. Use of POCUS may be limited by time and staffing constraints in the busy emergency department. However, unlike the standard workflow of traditional consultative ultrasonography, the EM POCUS examination may be limited in scope and goal directed; or, depending on the clinical situation, available time, and skill of the operator, it may be as comprehensive as the standard consultative examination.

### Comparison of EM and critical care POCUS

Because the EM physician and intensivist have shared responsibility for the care of the critically ill patient, they use ultrasonography in identical fashion for rapid evaluation of cardiopulmonary failure. The EM physician and the intensivist incorporate ultrasonography into the initial phase of management, while the intensivist uses it to guide the ongoing management once the patient is in the ICU. As a result, their applications of POCUS are identical in terms of the equipment requirements, image acquisition, image interpretation, and cognitive elements. However, EM and critical care ultrasonography (CCUS) differ in important ways (Table 1):

**Table 1:** Emergency department versus critical care ultrasonography

Critical care ultrasonography	Emergency department ultrasonography
Focused on cardiac, thoracic (pleura/lung), vascular diagnostic, screening abdominal, and procedural guidance [7]	Includes all critical care ultrasonography, extended abdominal, testicular, early obstetric, musculoskeletal, and ocular [4]
Initial and serial examinations for ongoing diagnosis and management	Typically single examination for diagnosis and disposition
Does not lead to decision to discharge from hospital	Frequently leads to decision to discharge from hospital

1. The EM physician typically uses POCUS in a more extended fashion than the intensivist to include advanced abdominal, obstetric, testicular, musculoskeletal, and ocular ultrasonography. This requires more extensive training than that needed by the intensivist, who generally focuses on cardiac, thoracic, screening abdominal, and venous ultrasonography.
2. The intensivist uses POCUS in the ICU as a diagnostic and monitoring tool, but does not use the examination results to discharge the patient from the hospital. The intensivist has the option of serial reassessment of the patient and may escalate to standard consultative imaging if required. In the emergency department, the final disposition of the patient may be determined by the ultrasonographic evaluation; POCUS performed by EM physicians is therefore a definitive evaluation when it leads to patient discharge from the hospital. However, when doubt or uncertainty exists regarding ultrasonography findings, EM physicians must consider the services of traditional consultative ultrasonography prior to final diagnosis, treatment, and disposition. The authors emphasize the need to call for assistance and advice from a more experienced operator in the presence of uncertain findings.

### Development of EM POCUS in North America and Europe

The American College of Emergency Medicine (ACEP) issued a position paper in 1990 [1] that supported the use of POCUS; this was followed by a similar document written by the Society for Academic Emergency Medicine in 1991 [2]. With this early support for the use of POCUS by EM physicians, EM residency programs in the United States and Canada started to introduce ultrasonography as a standard part of training. The ACEP developed an ultrasonography working group in the 1990s who were vigorous proponents of POCUS and were responsible for establishing POCUS as a widely accepted standard within the EM community. This required resolution of conflicts with the radiology and cardiology services related to jurisdiction, economics, and scope of practice. In 2001, the ACEP developed emergency ultrasound guidelines [3] that described the scope of practice for EM POCUS to include seven ultrasound competencies: trauma, pregnancy, abdominal aorta, cardiac, biliary, urinary tract, and procedural. These were expanded in 2009 to include thoracic, deep vein thrombosis (DVT), ocular, and soft tissue/musculoskeletal [4]. The American College of Graduate Medical Education (ACGME) has established POCUS as a required part of EM training based upon the recommendations of the professional societies. All EM residencies accredited by the ACGME provide POCUS training guided by consensus recommendations published by the ACEP in 2009 that include a minimum 80 hours of dedicated clinical ultrasonography, 20 hours of didactic ultrasonography education, and accurate performance of 150 independently reviewed ultrasound studies [5]. Many residencies exceed these minimum requirements; as a result, training in EM POCUS is now standard in the 190 EM training programs in the USA. There are also 95 EM ultrasonography fellowship training programs in the USA which provide an optional year of further training following standard residency training in EM for those physicians who seek special qualification in EM POCUS. The ACEP has developed guidelines for fellowship training [6] that include the performance of a minimum of 1000 ultrasonography examinations and 20 hours per month of dedicated ultrasonography practice, education, or research. The fellowship programs have been instrumental in filling the need for qualified faculty to provide training in POCUS to EM residents. At present, there is no nationally recognized certification in EM POCUS. Although this is controversial, the present consensus is that the requirement for a specific certification for POCUS is not necessary. Completion of residency training in EM by established ACGME standards implies competence in a wide variety of skills, none of which require specific certification. Ultrasonography should be seen no differently than other aspects of EM training. This is similar to the situation regarding certification in CCUS.

The development of POCUS in Europe is more difficult to summarize, because there is no central authority such as the ACGME that determines scope of practice or training standards. The development of EM POCUS has therefore been country specific. It is apparent to North American observers that EM POCUS is used in many hospitals in Europe, and that much of the best quality research related to POCUS comes from these centers of excellence. Lacking a central control of residency training requirements, the authors cannot comment on residency or fellowship training patterns in Europe.

### Scope of practice for EM POCUS

The EM physician is tasked with the initial evaluation and management of the patient with cardiopulmonary failure. Use of EM POCUS is an essential tool in this process, just as it is for the intensivist who provides follow-through care. The EM physician and intensivist share the same skill set as defined in the American College of Chest Physicians/La Societe de Reanimation de Langue Francaise (ACCP/SRLF) Statement on Competence in Critical Care Ultrasonography [7]. The only difference between the two specialties is that the intensivist uses CCUS for subsequent management in the ICU, whereas the EM physician uses it for initial management in the emergency department. The key components of CCUS are discussed as follows.

### Goal-directed echocardiography

The goal-directed echocardiography (GDE) examination uses a limited number of standard echocardiography views in order to allow the EM physician to rapidly assess cardiac anatomy and function in the patient with hemodynamic failure [8]. As defined in the ACCP/SRLF Competence Statement, the five standard views include the parasternal long-axis, parasternal short-axis, apical four-chamber, substernal, and inferior vena cava (IVC) views. Color Doppler analysis of the mitral and aortic valves may be included in the examination. The examination can be performed in a few minutes, and is generally combined with other aspects of CCUS to provide a whole-body ultrasonography (WBU) approach to the critically ill patient. The GDE examination has several purposes:

1. Identification of an immediately life-threatening cause for hemodynamic failure. The use of GDE permits prompt identification of an imminently life-threatening process where intervention may be life-saving such as major valve failure, pericardial tamponade, severe reduction in left ventricular function, or massive pulmonary embolism (PE). Although uncommon, these possibilities mandate early GDE for the patient in shock.
2. Categorization of shock state and initial management strategy. The five views of GDE permit the intensivist to categorize shock as a hypovolemic, distributive, cardiogenic, or obstructive pattern. This allows logical management strategies as well as identification of the cause of the hemodynamic failure.
3. Identification of coexisting diagnoses. The critically ill patient may have multiple diagnoses predating the hemodynamic failure or occurring as another acute process. The GDE examination may identify these other diagnoses that complicate the treatment of the primary process.

It well established that EM physicians can become competent in GDE [9–11]. Its use is supported by the professional societies of emergency and critical care medicine and by the American Society of Echocardiography [12]. The diagnostic utility of GDE has been well validated for the evaluation of undifferentiated shock [13–15], and GDE is useful for identification of potentially life-threatening processes that are not apparent on initial evaluation of the patient with shock [16]. GDE is productively combined with thoracic ultrasonography for the evaluation of respiratory failure [17].

### Ultrasonography in cardiac arrest

The subcostal long-axis view of GDE has utility for evaluation of cardiac arrest. The examination is performed during brief pulse checks when chest compressions are halted. Echocardiography is useful during cardiopulmonary resuscitation (CPR) for three purposes:

1. Identification of potentially reversible causes of cardiac arrest such as a large pericardial effusion with tamponade, a severely dilated right ventricle with acute cor pulmonale related to a PE, or a heart that is profoundly hypovolemic.
2. Identification of cardiac contractile activity without palpable pulse. Echocardiographic imaging during CPR allows reclassification of some patients who are clinically classified as having pulseless electrical activity, because even very weak endogenous cardiac contractility can be observed sonographically. The prognosis for return of spontaneous circulation is improved when there is some echocardiographic evidence of endogenous myocardial contractility, and echocardiography has been applied in prehospital settings to assess this [18].
3. Identification of the absence of cardiac contractile activity. In the patient who is receiving CPR in the emergency department, complete absence of cardiac contractile activity is a strong indicator that the resuscitation effort will not be successful [19–21].

### Thoracic ultrasonography (lung and pleural)

Ultrasonographic examination of the thorax allows the EM physician to rapidly assess the patient with respiratory failure for normal aeration pattern, pneumothorax, lung interstitial syndrome (LIS), consolidation, or pleural effusion [22]. The examination can be performed quickly and is a key component of the WBU approach for the patient with cardiopulmonary failure. When compared with standard radiography, thoracic ultrasonography is superior for the characterization of abnormalities that are relevant to the assessment of respiratory failure such as pneumothorax, pneumonia, pleural effusion, and alveolar/interstitial diseases [22–25]. Given the intrinsic problems of chest radiography in the critically ill patient (anterior–posterior projection, rotation, penetration artifacts), it is possible that thoracic ultrasonography could be the primary imaging modality for evaluation of respiratory failure in the emergency department and ICU [26]. Thoracic ultrasonography has similar performance characteristics to chest computed tomography (CT) when assessing the aforementioned abnormalities, and addition of thoracic ultrasonography improves diagnostic accuracy and efficiency in the emergency department [27].

Using thoracic ultrasonography, EM physicians may reliably differentiate between patients with acute decompensated heart failure [17, 23, 28], pneumonia [24, 29], acute respiratory distress syndrome [30], pneumothorax [25], PE [31, 32], and diaphragmatic dysfunction [33, 34]. Decompensated heart failure and cardiogenic pulmonary edema may be differentiated from noncardiogenic causes of dyspnea with sensitivity and specificity of 94 % and 92 % respectively [23]. Lung ultrasonography has 94 % sensitivity and 96 % specificity for pneumonia [29], and has 91 % sensitivity and 98 % specificity for pneumothorax [25]. Visual representation of lung ultrasonography semiology has been combined with thoracic ultrasonography protocols for rapid and accurate diagnosis of pulmonary disease and to guide fluid resuscitation [35, 36].

### Examination for DVT and PE

Examination for DVT is a key part of the WBU approach to cardiopulmonary failure, particularly if PE is a consideration. EM physicians can perform high-quality 2-D venous compression studies with results similar to those performed by the consultative radiology service [37] while avoiding the inevitable delay required to obtain a radiology study [38]. Doppler-based measurements do not add to the yield of the 2-D compression study [39], so the POCUS DVT examination can be performed rapidly [40].

While a positive DVT study has immediate implication in the patient who is being evaluated for PE, a negative DVT study does not rule out PE. If the rest of the WBU examination reveals another cause for the symptom complex (e.g., pneumonia, congestive heart failure), it is very unlikely that the patient has a PE [31]. If lung ultrasonography reveals findings consistent with PE (e.g., small subpleural consolidations in lower lobes), it is a high-probability consideration. Ultrasonography may reduce the need for CT angiography for the detection of PE [32], but some patients still require CT if ultrasonography is indeterminate.

### Screening abdominal ultrasonography

Given the complexity of abdominal ultrasonography, the ACCP/SRLF Competence Statement suggests the POCUS approach should be limited to identification of intrabdominal fluid, examination of the aorta, and assessment for hydronephrosis or bladder distention in critical care settings. The intensivist typically turns to the consultant radiologist for more advanced evaluation of the hepatobiliary tree, but the EM physician may choose to develop more advanced capability than the intensivist, particularly related to evaluation of the right upper quadrant (vide infra).

Ultrasonography evaluation of the aorta includes shape and caliber as well as inspection for a dissection flap. Transthoracic and transabdominal ultrasonography cannot be utilized in isolation to rule out aortic dissection; however, visualization of an intimal flap with differential Doppler flow does have high specificity for dissection [41]. Bedside ultrasonography for abdominal aortic aneurysm (AAA) is included in many algorithms evaluating patients in shock [15], because visualization of a normal caliber aorta precludes ruptured AAA as a diagnosis. Emergency physicians are able to identify AAA with sensitivity of 99 % and specificity of 98 % [42], enabling early diagnosis of ruptured AAA.

Renal ultrasonography assists the EM physician in the management of acute kidney injury, urinary tract infection, and nephrolithiasis. Absence of hydronephrosis rules out obstructive nephropathy and obstructed urinary tract infection requiring procedural drainage for source control [43]. While renal ultrasonography rarely diagnoses nephrolithiasis in the emergency department, a normal sonogram categorizes patients with renal colic as low risk for complications. There is no difference in complications, pain, return visits, admissions, or diagnostic accuracy between patients with suspected nephrolithiasis when evaluated with POCUS, radiology ultrasonography, or CT [44]. Ultrasonography allows the EM physician to quickly and efficiently provide a disposition for patients with renal colic while avoiding unnecessary radiation exposure.

### Ultrasonography in trauma

Identification of free fluid has application for the EM physician during the initial evaluation of the patient with thoracic or abdominal trauma. The focused assessment with sonography in trauma (FAST) examination utilizes ultrasonography to identify an intrabdominal source of bleeding. FAST has replaced peritoneal lavage as the technique of choice for evaluation of abdominal trauma and is standard practice [45]. The FAST examination can be performed rapidly. If initially negative, the FAST can be repeated as clinically indicated. In the presence of hemorrhagic shock, a positive examination indicates intrabdominal bleeding and the need for procedural or operative management.

The extended FAST examination is performed in patients with thoracic trauma. The examination includes the subcostal view of the heart along with the anterior and lateral chest. A pericardial effusion in a patient with thoracic trauma and hemodynamic compromise requires consideration of pericardial tamponade and immediate decompression. Ultrasonography of the anterior and lateral chest identifies pneumothorax and hemothorax with greater sensitivity and specificity than supine chest radiography [46] and may replace chest radiography for this purpose [47]. The CT scan remains the gold standard for diagnosis of pneumothorax and hemothorax; however, in comparison, POCUS gives similar results and offers the advantage of being a point-of-care technique. Ultrasonography is superior to standard supine radiography for detection of hemothorax [48]. While body CT is the primary imaging tool for evaluation of the trauma patient, POCUS remains the best initial modality for the emergency evaluation of abdominal and thoracic trauma. Ultrasonography provides rapid identification of imminently life-threatening injuries requiring immediate intervention. Body CT may then follow. Bedside ultrasonography in trauma is performed more rapidly than CT and is recommended by the ACEP for patients with blunt thoracic or abdominal trauma [49].

### Ultrasonography for procedure guidance

Ultrasonography is used by the EM physician for guidance of a variety of procedures that are required for treatment of critical illness or routine management of the disease process [50, 51]. Ultrasonography increases the success rate and reduces the complication rate of a wide variety of procedures that are performed by EM physicians and intensivists, such as thoracentesis (both diagnostic and therapeutic requiring chest tube insertion), paracentesis, regional anesthesia, lumbar puncture [51], central venous catheter insertion (at all sites [52, 53]), difficult peripheral arterial and venous catheter insertion [50, 54], incision and drainage of cutaneous abscess [55], arthrocentesis [56], and airway management [57].

### Use of EM POCUS for noncritical care applications

The intensivist and the EM physician share common ground when using CCUS. However, the EM physician extends the scope of practice to include a variety of applications that are beyond the competence level of the intensivist.

### Abdominal ultrasonography

Ultrasonography of the right upper quadrant with POCUS is useful for evaluation of acute cholecystitis. Ultrasonography for acute cholecystitis performed by EM physicians is as accurate as consultative radiology ultrasound and cholescintigraphy [58, 59]. Utilization of POCUS for cholecystitis allows EM physicians to localize the source of occult sepsis and allows a means to efficiently treat and provide a disposition for their patients.

Diagnosis of appendicitis may be made with POCUS. Visualization of the appendix by ultrasonography is technically difficult and dependent on patient body habitus and cooperation with the examination. The appendix may be obscured by overlying bowel gas, so nonvisualization of the appendix is a nondiagnostic study. When the appendix is visualized, ultrasonography has sensitivity of nearly 100 % and specificity of 80–90 % [60, 61]. Rates of appendix visualization are increased for patients with low body mass index (<22), higher pain scales (>6), and higher Alvarado scores (>6) [62]. Pediatric and pregnant patients with appendicitis are more likely to meet these criteria, and ultrasonography may be used as the sole imaging for these patients.

### Ultrasonography in obstetrics

Pelvic pain is a common presenting symptom in the emergency department, and its evaluation with ultrasonography has been reviewed in the literature [63]. In early pregnancy, the primary concern for patients with pelvic pain is ectopic pregnancy. The ACEP supports the use of POCUS by EM physicians for this indication [4]. Ultrasonography performed by EM physicians has 99 % sensitivity and a 99.9 % negative predictive value for ectopic pregnancy [64]. Utilization of both abdominal and transvaginal ultrasonography (TVUS) in the emergency department is safe, accurate, and reduces patient length of stay [65, 66]. Emergency physician TVUS also provides prognostic information for pregnant patients with vaginal bleeding and indeterminate ultrasonography findings [67]. Utilizing TVUS allows EM physicians to rapidly diagnose life-threatening emergencies while providing accurate and efficient dispositions for patients at low risk.

The ACEP does not include pelvic ultrasonography as a recommended competency for EM physicians outside of its application in early pregnancy. The diagnosis, treatment, and disposition of nonpregnant patients with pelvic pain often require advanced imaging capabilities provided by the radiology service.

### Testicular ultrasonography

Ultrasonography performed by EM physicians has sensitivity of 96–100 % and specificity of 80–90 % for the diagnosis of testicular torsion, and has sensitivity and specificity of 80–90 % for epididymo-orchitis [68, 69]. Many emergency departments do not have ready access to consultative radiology ultrasonography during all hours of operation. Ultrasonography for testicular torsion can be performed successfully by emergency physicians [70] and may improve outcome in torsion where rapid diagnosis leads to increased rates of testicular salvage.

### Soft tissue and musculoskeletal ultrasonography

Soft tissue and musculoskeletal ultrasonography is a relatively new application of POCUS and includes fracture identification, evaluation of tendon injury, and foreign body identification. Ultrasonography may diagnose some fractures with sensitivity >90 %, including injuries which are difficult to diagnose with conventional radiography such as scaphoid fractures [71]. Screening with ultrasonography for fractures may reduce the need for radiography, facilitating efficient treatment and disposition of patients with low-acuity injuries [72]. Ultrasonography by EM physicians has sensitivity of 100 % and specificity of 95 % for extremity tendon injuries [73]. It is more sensitive than physical examination for detection of partial tendon injuries; and reduces costs, time requirements, and morbidity when compared with magnetic resonance imaging or surgical exploration. Foreign body identification is facilitated by POCUS. Radiographic identification of radiolucent materials such as glass or wood is limited. Ultrasonography may identify these foreign bodies, allowing targeted removal and a reduction in repeat visits, patient morbidity, and costs [74].

### Ocular ultrasonography

Ocular ultrasonography is another relatively new application for EM physicians endorsed by the ACEP [4]. Reviews of anatomy, technique, and pathology are available [75]. Emergency physicians are able to accurately evaluate globe rupture, foreign bodies, retinal detachment, lens dislocation, and vitreous hemorrhage or detachment with ultrasonography [76]. This allows patient stratification into emergent, urgent, and routine ophthalmologic consultation.

Ocular ultrasonography may be used to evaluate intracranial pressure (ICP) by measurement of the optic nerve sheath diameter. At a cut-off value of 5 mm, ultrasonography has 100 % sensitivity for evaluation of increased ICP when compared with CT and has 84 % sensitivity for any intracranial injury in the setting of head trauma [77, 78]. Emergency physicians are able to accurately measure the optic nerve sheath diameter [79], and this measurement is highly correlated with direct ICP measurements with sensitivity and specificity >90 % [80, 81]. Optic nerve sheath measurements reliably and rapidly change with changes in ICP [82].

## Conclusions

Ultrasonography has become an integral part of EM over the past two decades. Some aforementioned applications of ultrasonography are well established and practiced routinely, while more research is necessary to advance the use of ultrasonography in other areas.

## Abbreviations

AAA, abdominal aortic aneurysm; ACCP/SRLF, American College of Chest Physicians/La Societe de Reanimation de Langue Francaise; ACEP, American College of Emergency Physicians; ACGME, American College of Graduate Medical Education; CCUS, critical care ultrasonography; CPR, cardiopulmonary resuscitation; CT, computed tomography; DVT, deep vein thrombosis; EM, emergency medicine; FAST, focused assessment with sonography in trauma; GDE, goal-directed echocardiography; ICP, intracranial pressure; IVC, inferior vena cava; LIS, lung interstitial syndrome; PE, pulmonary embolism; POCUS, point-of-care ultrasonography; TVUS, transvaginal ultrasonography; WBU, whole-body ultrasonography
